# Eccrine syringofibroadenoma: report of two exuberant cases^☆^

**DOI:** 10.1016/j.abd.2022.10.017

**Published:** 2024-02-24

**Authors:** Camila Schlang Cabral da Silveira, Luiz Felipe Oliveira Santos, Marcella Leal Novello D’Elia, Daniel Lago Obadia

**Affiliations:** Department of Dermatology, Hospital Universitário Pedro Ernesto, Universidade do Estado do Rio de Janeiro, Rio de Janeiro, RJ, Brazil

Dear Editor,

Eccrine syringofibroadenoma (ESFA) is a rare benign adnexal tumor that arises from the excretory portion of the eccrine sweat glands.^1^ It usually involves distal extremities in middle-aged to elderly patients,^1^ presenting as solitary or multiple, coalescent, firm, skin-colored verrucous nodules, of variable sizes.^1^ It is currently classified into five types: solitary lesions, multiple lesions associated with ectodermal dysplasia, lesions without additional cutaneous pathology, nevoid lesions and reactive lesions.^2^ Clinical diagnosis is very difficult and, therefore, histopathological evaluation is essential.^1^ Complete excision seems to be the definitive treatment.^2^ The present report describes two cases of ESFA with an exuberant clinical presentation and diagnostic difficulty due to limited access to specialized services, albeit with excellent final results after excision by shaving.

## Case 1

**Figure 1:**
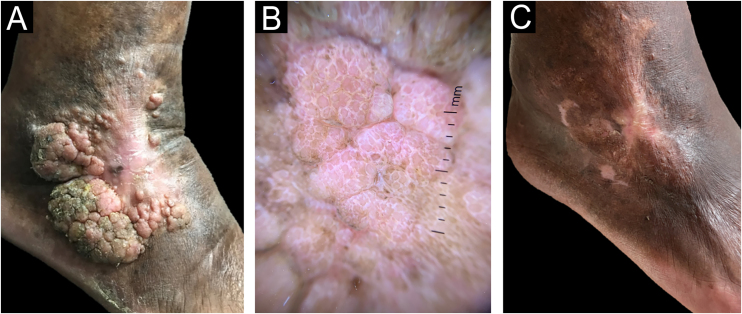
Case 1. (A) Prior to treatment. (B) Dermoscopy: pink lacunae separated by white septa. (C) Outcome four months after excision by shaving.

**Figure 2:**
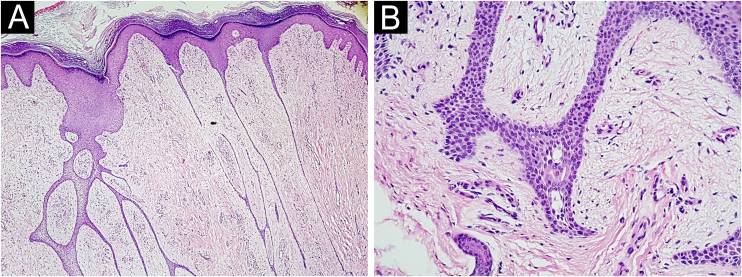
Histopathology of Case 1 stained with Hematoxylin & eosin. (A) ×20 magnification, showing a network of epithelial cords connected to the epidermis. (B) At ×200 magnification, the fibrovascular stroma is observed between the epithelial cords.

**Figure 3:**
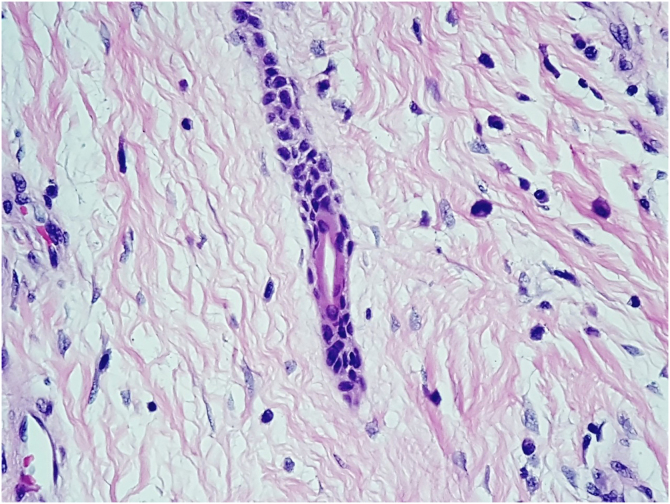
At ×400 magnification. Eccrine duct lumen inside the epithelial cord.

A 76-year-old hypertensive diabetic male patient was referred to the dermatology service due to a vegetative lesion around a painful ulcer on the right lateral malleolus that had been noticed in 2018. He denied triggering factors and reported a previous biopsy at an external service diagnosed as verruca vulgaris. Associated with the condition, he had lymphedema of the ipsilateral lower limb. He underwent treatment for the ulcer but showed progression of the vegetative lesion. On examination, there was a hypertrophic scar on the lateral region of the dorsum of the right foot and multiple hardened verrucous pink nodules measuring 9 cm in their largest diameter, which coalesced on the lower edge of the scar (Fig. 1A). Dermoscopy showed no findings suggestive of malignancy (Fig. 1B). A new biopsy was performed, followed by complete removal of the lesion, by shaving, after histopathological evaluation. The image (Fig. 1C) shows the four-month postoperative period, without recurrence. Histopathology disclosed thin cords of cuboidal epithelial cells anastomosed in a network-like fashion in connection with the lower portion of the epidermis (Fig. 2A). A fibrovascular stroma was observed interspersing the epithelial cords (Fig. 2B). The cells that constituted the cords showed ductal differentiation (Fig. 3); findings compatible with ESFA. There was no evidence of malignant transformation.

## Case 2

**Figure 4:**
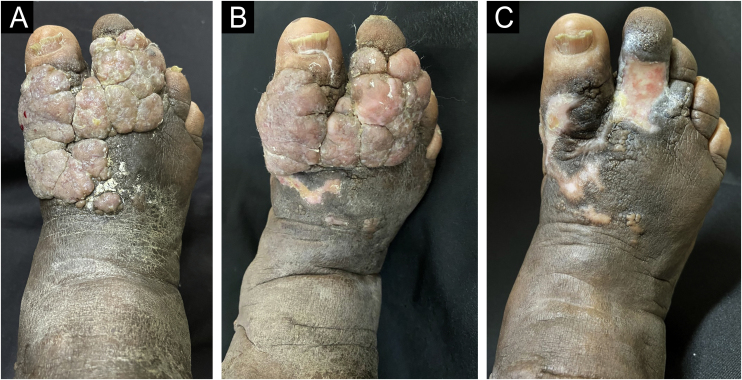
Case 2. (A) Prior to treatment. (B) One month after the first shaving resection in the proximal region of the lesion. (C) One month after complete excision.

**Figure 5:**
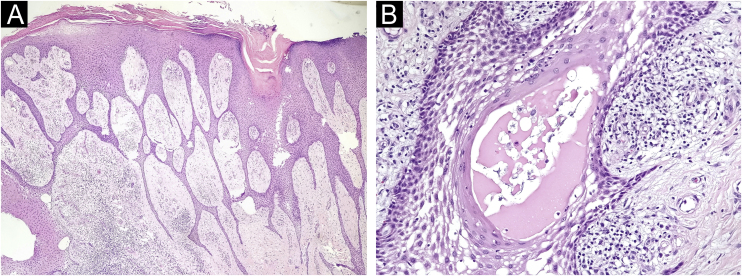
Histopathology of Case 2, stained with Hematoxylin & eosin. (A) ×20 magnification, showing a network of epithelial cords connected to the lower portion of the epidermis. (B) ×400 magnification, showing eccrine duct lumen inside an epithelial cord.

A 61-year-old female diabetic patient was referred to the dermatology service due to the appearance of a vegetative lesion six years previously. She reported that the condition began as papular lesions that developed into a tumor on the dorsum of the right foot. On examination, there was a vegetative lesion measuring approximately 10 cm in its largest diameter, well demarcated, with serosanguineous exudate and a foul odor, associated with hardened edema and hyperchromia of the distal third of the limb (Fig. 4A). She denied triggering factors. An incisional biopsy was performed followed by serial shaving of the lesion until its complete excision, and healing by secondary intention (Fig. 4B and C), after histopathological confirmation of ESFA (Fig. 5 A and B). All surgical specimens were sent for pathological analysis, to exclude the possibility of malignant transformation.

## Discussion

ESFA is a rare benign neoplasm that normally presents as a single, nodular, large asymptomatic plaque, but which can be multiple, coalescent, firm, skin-colored and verrucous in appearance at the margin of an ulceration.^1^ Among the findings of the physical examination, a possible characteristic of the affected region is the “mossy leg” aspect,^3^ a characteristic observed in the reported cases. The lesion has a predilection for distal extremities but can affect other sites.^4^ The origin is not well defined, but it is associated with the proliferation of adnexal epithelial cells that form ducts,^5^ which arise from the excretory portion of the eccrine sweat glands.^4^

ESFA is divided into five types according to morphology, number of lesions and associated factors.^1^ The solitary subtype is the most common,^4^ represented by the appearance of a verrucous mass or single non-hereditary nodule located on the lower limbs of elderly patients.^6^ This description seems to fit the patient in the second case report, who had only one lesion on the lower limb, and no association with previous trauma or heredity. The first patient had a chronic ulcer, difficult to heal, was diagnosed with diabetes and lymphedema, suggesting the reactive subtype, which typically affects the acral region and is secondary to a chronic inflammatory or neoplastic lesion, having been previously described in association with ulcers, lymphedema and diabetic foot.^1^, ^6^ A specific type of eccrine remodeling or ductal repair, due to repeated damage to eccrine structures, is believed to be the pathogenesis.^1^, ^6^

Histopathology is essential for diagnosis.^2^ The formation of thin anastomosed epithelial cords, consisting of benign cuboidal epithelial cells with ductal differentiation, creates a network that connects with the lower portion of the epidermis;^3^ these cells are basaloid and smaller than the adjacent keratinocytes.^3^ Rich fibrovascular stroma can be observed between the cords, containing plasma cells and ductal structures.^6^ There may or may not be lumen formation and discrete lymphocytic infiltrate.^3^ Immunohistochemical analysis shows positivity with epithelial membrane antigen,^6^ carcinoembryonic antigen (CEA)^7^ and CK19, which identifies ductal differentiation.^3^

Some authors state that malignant transformation may occur in long-standing ESFA.^5^ Areas of malignancy can easily be missed in incisional biopsies; therefore, complete excision is the treatment of choice,^6^ especially in cases of solitary lesions.^1^ In the two reported cases, it was decided to perform excision by shaving and regular outpatient monitoring of the lesion bed.

## Conclusion

The relevance of the cases lies in the rarity of the disease, especially exuberant ones. Both cases showed a delay in the diagnosis, in the first due to a divergent histopathological diagnosis and in the second, due to difficulty in accessing a service capable of carrying out the investigation. Knowledge about the disease and diagnostic possibilities, considering the clinical presentation, helps to guide investigation and treatment more appropriately.

## Financial support

None declared.

## Authors’ contributions

Camila Schlang Cabral da Silveira: Drafting and editing of the manuscript; design and planning of the study; collection, analysis and interpretation of data; intellectual participation in the propaedeutic and/or therapeutic conduct of the studied cases; critical review of the literature; approval of the final version of the manuscript.

Luiz Felipe Oliveira Santos: Drafting and editing of the manuscript; collection, analysis and interpretation of data; intellectual participation in the propaedeutic and/or therapeutic conduct of the studied cases; critical review of the literature; approval of the final version of the manuscript.

Marcella Leal Novello D’Elia: Drafting and editing of the manuscript; design and planning of the study; collection, analysis and interpretation of data; intellectual participation in the propaedeutic and/or therapeutic conduct of the studied cases; approval of the final version of the manuscript.

Daniel Lago Obadia: Drafting and editing of the manuscript; design and planning of the study; collection, analysis and interpretation of data; intellectual participation in the propaedeutic and/or therapeutic conduct of the studied cases; approval of the final version of the manuscript.

## Conflicts of interest

None declared.
